# Struggling in no-man’s land between childhood and adulthood – a phenomenological-hermeneutical video-observation study exploring adolescent males’ encounters with general practitioners

**DOI:** 10.1080/02813432.2025.2475507

**Published:** 2025-03-10

**Authors:** Johanna Haraldsson, Linus Johnsson, Per Kristiansson, Ylva Tindberg, Lena Nordgren

**Affiliations:** a Department of Public Health and Caring Sciences/Family Medicine and Preventive Medicine, Uppsala University, Uppsala, Sweden; b Centre for Clinical Research Sörmland, Uppsala University, Eskilstuna, Sweden; c Department of Public Health and Caring Sciences/Centre for Research Ethics & Bioethics, Uppsala University, Uppsala, Sweden; d Department of Women’s and Children’s Health, Uppsala University, Uppsala, Sweden; e Department of Public Health and Caring Sciences/Caring Sciences, Uppsala University, Uppsala, Sweden

**Keywords:** General practitioners, family practice, physician-patient relations, adolescent medicine, qualitative research, adolescents, Sweden

## Abstract

**Objective:**

Many adolescent males report negative experiences of consultations with general practitioners (GPs), which contrasts with the importance of patient-centredness that GPs themselves emphasise. A better understanding of this discrepancy might facilitate improvements. The aim was to explore and describe adolescent males’ encounters with GPs in Swedish primary healthcare centres using a lifeworld perspective.

**Design:**

Qualitative lifeworld-based study. Video-recorded observations were analysed using a phenomenological-hermeneutical method.

**Setting:**

Two primary healthcare centres in mid-Sweden.

**Subjects:**

Nine males aged 15 to 19, video-recorded during their encounters with GPs in March through May 2022.

**Findings:**

Adolescent males navigate between being children in need of parental support and men who can take initiative and responsibility. They face cognitive, emotional, and relational demands, the complexity of which renders them particularly vulnerable. When feeling exposed and not knowing what to expect, they struggle to make themselves understood, and to understand what the GP is saying and what is happening. The difficulties that they have disclosed to the GP in trust need to be recognised and carefully acted upon. Thus the GP must respond appropriately to this complex mix of vulnerabilities to prevent feelings of disappointment or of having exposed themselves in vain.

**Conclusion:**

The complexity of encounters with adolescent males imposes great demands on GPs to identify and adapt to their individual needs. A proper ethical response involves helping them navigate the challenges of the consultation while also respecting them as persons and meeting their age-dependent needs.

## Introduction

One of the most important factors that influence adolescents who consider consulting a general practitioner (GP) is whether they have perceived prior encounters as helpful [1–3]. Although many adolescents want to discuss their concerns regarding lifestyle and mental health [4,5], there is a risk that such problems will not be brought up to discussion with the GP [5]. This is a problem particularly among adolescent males, whose overall mortality is twice that of their female peers, mostly due to preventable issues related to lifestyle and mental health [6,7]. Male adolescents use less healthcare than their female counterparts [8–10] and rarely consult for mental health issues [11–13]. However, those who do occasionally consult a GP have been found to engage more frequently in health-compromising behaviours compared to less frequent visitors [14,15]. More importantly, in many studies adolescents describe their encounters with GPs using negative language, depicting them as dismissive, uncompassionate, and untrustworthy, and recount experiences of not being listened to or not taken seriously [1,13,16]. These findings contrast with the person-centredness and responsiveness to their patients’ needs that GPs themselves emphasise as core values [17,18]. Since GPs may not be fully aware of how their communication is experienced, what they in fact do might not always match what they claim to be doing [18,19]. Disparities such as these can be revealed by observing doctor-patient interactions [20,21].

To explain the discrepancy between adolescent males’ reported experiences and GPs’ intentions, a deeper understanding of what actually happens in these encounters is necessary. This can be achieved through qualitative observations based on a lifeworld perspective [20,22]. The lifeworld perspective aims to illuminate meanings of lived experience [23], such as adolescent males’ experiences of encounters with GPs. According to the lifeworld theory, first described by Husserl, we all perceive our shared world differently due to variations in culture, education, or other disparities [22,23]. The lifeworld is our lived reality, the world we know through our lived experiences [22,23]. Qualitative observations can reveal new insights into adolescent males’ lived experience of encounters with GPs by allowing context, actions, and non-verbal communication to be included in the analysis [20]. Non-verbal communication such as gestures, posture, or facial expressions constitute important parts of the communication and can affect the experience of the encounter [24,25]. As far as we know, adolescent males’ encounters with GPs have never been studied using video-recorded qualitative observations.

Any good consultation with a GP, even for a simple medical issue, can make the adolescent male more likely to seek help from health care in the future [1,2], thus promoting their future health. Hence, the aim of this study was to explore and describe adolescent males’ encounters with GPs in Swedish primary healthcare centres using a lifeworld perspective.

## Methods

### Design and setting

Since the aim was to explore and describe encounters, a qualitative research approach was chosen. The study is part of a larger project aiming to illuminate adolescent males’ encounters with GPs through video observations and interviews. This paper is based only on the video observations.

The study was conducted in a primary care context in mid-Sweden in 2022. Data were collected through video recordings at two public healthcare centres. Healthcare centre A was located in a small town with demographics approximately similar to Sweden as a whole [26]. Healthcare centre B was located in a medium-sized town with higher rates of unemployment, a larger population born outside Sweden, and fewer adolescents completing compulsory education with good enough grades to be eligible for upper secondary school [26].

### Participants

All males born between 2002 and 2006 who, for any reason, visited any of the GPs (described below) involved in the project were considered eligible. Other healthcare staff briefly informed the adolescent males about the study. Those who agreed to be contacted by the researchers were telephoned by JH a few days before their appointment and invited to participate or, in the case of urgent appointments or when unreachable by phone, on arrival at the healthcare centre. Exclusion criteria were urgent and serious illness (e.g. those requiring ambulance transport) and inability to communicate in Swedish without an interpreter; three adolescent males were excluded due to the latter. The number of participants was not decided beforehand; instead, inclusion ended when sufficient information power was achieved through the collection of rich and varied data [20,27].

**Figure 1.**
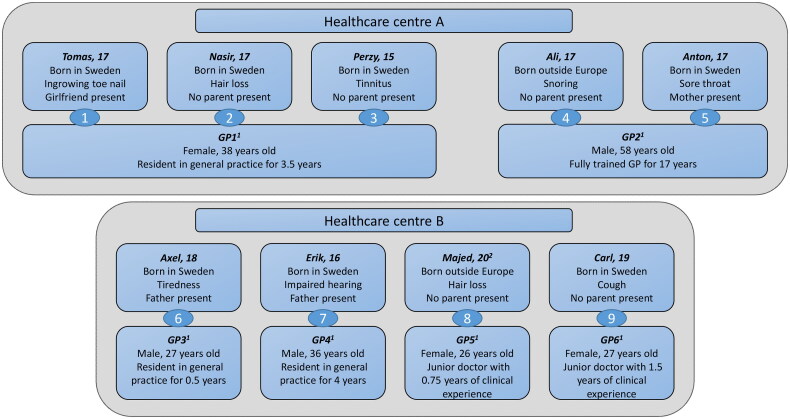
Characteristics of participating adolescent males (age, origin, reason for visiting, and whether accompanied) and GPs (sex, age, and clinical experience). Nine adolescent males visited six different GPs, yielding nine video-recorded encounters. At healthcare centre A, Tomas, Perzy, and Nasir visited GP1, and Ali and Anton visited GP2. At healthcare centre B, Axel visited GP3, Erik GP4, Majed GP5, and Carl GP6. The adolescent males chose their pseudonyms themselves, but some of them have been adjusted in order to better protect their privacy. ^1^GPs: general practitioners. ^2^Majed, who had recently turned 20, was included as a result of using year of birth as inclusion criterion instead of actual age.

Nine out of fifteen invited adolescent males agreed to participate: five at healthcare centre A and four at healthcare centre B (mean age 17.3 years), yielding nine video-recorded encounters (Figure 1). Five adolescent males met with the GP alone, while three were accompanied by a parent and one by his girlfriend. Two were born outside Sweden, which is representative of Sweden as a whole (Figure 1) [28].

All medical doctors (about 25) working as GPs in either of the two healthcare centres were considered eligible. They included fully trained GPs (in Swedish: *specialist i allmänmedicin*), residents in general practice (*ST-läkare*), and junior doctors, as long as they worked as and would be perceived as GPs by visiting adolescent males. For simplicity, they are all hereinafter referred to as GPs. Eligible GPs were invited to participate during regular staff meetings held at each of the two healthcare centres. Fourteen GPs, five at healthcare centre A and nine at healthcare centre B, agreed to participate.

Three of the adolescent males visited GP1, two visited GP2, and the remaining four visited four different GPs. Anton had seen GP2 once before. None of the others were familiar with the GP they visited. Consequently, only six of the fourteen volunteering GPs actually participated in the study. As a result of scheduling and appointment booking practices, these six GPs were younger and less experienced than those who did not participate (Figure 1).

### Ethical considerations

The use of video-recorded observations imposes a significant responsibility on the researchers to protect the participants’ privacy and dignity throughout the research process. The study design was approved by the Swedish Ethical Review Authority in Linköping (Dnr 2022-00075-01). Swedish law does not require parental consent for study participation by adolescents over 15 years [29]. Prior to participation, all participants received oral and written information about the study’s aim, the procedure, secure data storage, and privacy protection on arrival at the healthcare centre, whereafter they completed their consents. They received two cinema tickets each as a gift, which they had not been informed about beforehand.

### Data gathering

Video observations have been used previously to study communication in medical encounters [21,24,30,31]. Compared to direct observations, video recordings make it easier to capture events occurring simultaneously, thus facilitating observations of complex interactions [21].

Before each of the nine video-recorded encounters, the adolescent male and the GP jointly decided whether the researcher (JH) would be present. JH left the room after starting the video recording in all but one of the encounters. The camera was placed on a tripod on the floor and directed towards the patient’s and the GP’s chairs, while keeping the examination couch out of frame. The GP was instructed to put the lens cap on or to turn the camera away if necessary. In three cases the physical examinations were recorded with the adolescent male sitting fully dressed in the patient’s chair. JH wrote field notes documenting contextual information, for example how the adolescent males interacted with their parents in the waiting area. After the consultation with the GP, the adolescent males were interviewed for other studies. One adolescent male asked not to appear on camera, but agreed to be audio-recorded.

### Analysis

The video-recorded encounters were analysed using a phenomenological-hermeneutical method in accordance with Lindseth and Norberg [22,32]. Phenomenological-hermeneutical research aims to illuminate meanings of lifeworld phenomena as they develop through time and can be used to explore meanings of lived experiences of phenomena [22]. This approach aims to make the reader aware of alternative ways to perceive reality and is thus applicable in studies that intend to improve healthcare [22].

Hermeneutic observation can be used to study clinical interactions [20] by uncovering experiences that are difficult to articulate and which would therefore otherwise remain invisible. It is less influenced by participants’ stated opinions than interviews, which makes the method suitable for exploring new perspectives on the deeper meanings of clinical encounters [20]. However, to observe is to interpret what one sees and otherwise perceives [20]. In this process of understanding, the observers’ preunderstanding is intrinsic [20]. The observers must, however, keep their judgements within brackets in order to remain open to what happens in the observed situation [22,32]. A sensitive openness to the phenomenon is a way of establishing validity, and can be described as a true willingness to see and understand, to expect the unexpected, and to avoid hasty conclusions [23].

Phenomenological hermeneutics comprises three interpretative arcs: naïve understanding, structural analysis, and comprehensive understanding [22,32]. It starts with concrete reflection, in which meaning is understood by putting what mattered in the situation into words [22]. It is a process of telling and retelling, until an expression that makes sense is found [22]. The analysis moves between the parts and the whole, and between explanation and understanding, in what is called the hermeneutic circle [22,32].

First, to gain a naïve understanding, the authors observed the video recordings with openness, letting themselves be touched by the content in order to grasp the meaning of the observed situations as a whole. They focused on how the adolescent males seemed to experience the encounter based on their verbal and non-verbal communication. Two, often three, authors watched the video recordings together. Notes were taken and reflected upon, and a short résumé of each video recording was written. JH synthesised the nine résumés, rewatched the video recordings, and rewrote the description until all authors agreed on an initial naïve understanding.

The second arc of interpretation, the structural analysis, was guided by the encounters’ intrinsic structure. While watching the video recordings, the authors realised that all nine encounters comprised three phases: the adolescent male describing his problem; the physical examination; the GP explaining what was wrong and what to do next. Since the examination couch was not visible on camera, in most cases we relied solely on audio for analysis of the examination phase. However, we were able to distinguish a number of helpful verbal cues, such as reflections made by the GP indicating that the adolescent male might be embarrassed, worried, or in pain. Two to four authors at a time watched each video recording phase by phase, reflected together on the content, and described what was taking place in short texts, one for each phase. All authors engaged in the analysis of the video recordings, albeit not at the same time. Excerpts from these short texts are presented in the results section. The texts were divided into meaning units, each labelled with a code [32]. For each phase, patterns in the texts and codes were searched for, and themes were developed [32]. While each code was restricted to a single phase, themes were allowed to span all three phases. Through discussions involving all the authors, the naïve understanding and the structural analysis were validated against each other in a circular process that enriched the understanding of both.

The third arc of interpretation consisted of a synthesis of the naïve understanding, the themes from the structural analysis, moral philosophical theory, and the authors’ preunderstanding into a comprehensive understanding [22,32], which was then further reflected upon to discover new aspects and gain a deeper understanding [32].

## Results

The naïve understanding and the structural analysis are presented in this section; the comprehensive understanding can be found in the discussion section. According to the phenomenological-hermeneutical method, which aims to discover the essential meaning of the phenomenon, the findings are stylistically presented from the perspective of the lived experience of an adolescent male [23,33,34]. The pronoun ‘He’ does not represent any specific participant but is used (with a majuscule) to distinguish the adolescent male from other male individuals.

### Naïve understanding

The encounter with the GP made the adolescent male tense and forced Him to concentrate. He made a great effort to explain His problem and to understand the doctor. He needed to decide to what extent the doctor could be trusted. He tried hard to answer the doctor’s questions adequately and keep up with the doctor’s pace. He had prepared Himself beforehand to be able to take on the part of an active participant who would take charge of His treatment and follow-up. He listened carefully and attentively to the doctor’s explanation and asked questions when He did not understand. Sometimes He trusted and accepted the doctor’s advice in the end despite already knowing what He wanted. He was clearly hopeful about getting better.

The encounter, however, also brought discomfort and exposure. Talking about His problems and His body was a potential source of embarrassment, as was having to undress and be examined. Outwardly, He might appear worried, watchful, and eager for help; or else reserved, seemingly unconcerned. He endured these challenges, trustingly or reluctantly. Being seen and acknowledged by the doctor would relieve the tension, but such an empathic response was not guaranteed.

Facing these challenges, the adolescent male would often need support. Parental support, however, could itself be challenging. Although parents might be helpful when trying to understand and answer the doctor, they could also take over the conversation or behave in an embarrassing manner. Even absent parents might impact the encounter by setting the agenda or by forcing Him to consider the risk of them finding out what He had disclosed.

### Structural analysis

One main theme and four themes were developed in the structural analysis. All themes were found in the three phases of the encounter, i.e. describing the problem, examination, and explanation. The main theme was called *Struggling in no-man’s land between childhood and adulthood*, and the four themes were called *Being strained by incomprehensibility*, *Mitigating and enduring vulnerability*, *Being respectfully seen and cared for,* and *Navigating initiative and responsibility* (Table 1). Apart from the main theme, the descriptions of the themes are structured according to the three phases of the encounter. Excerpts from the short descriptive texts used in the analysis are included to illustrate the themes.

**Table 1.** Presentation of how the main theme and the four themes manifest themselves through the three phases of the encounter.

		Phase of the encounter		
	Name of the theme	Describing the problem	Examination	Explanation
Main theme	Struggling in no man’s land between childhood and adulthood	He struggles to understand, not knowing what to expect. He tries hard to make Himself understood and to endure his vulnerability. He wants to take responsibility, while still needing support.		
Theme 1	Being strained by incomprehensibility	He concentrates intensely to explain well enough so that the doctor will understand.	He dreads what will happen. He cannot understand the findings in relation to His problem.	Trying to understand a long, complicated, and sometimes rushed explanation makes Him exhausted.
Theme 2	Mitigating and enduring vulnerability	He struggles to maintain His dignity, to guard His boundaries, and to avoid shame.	He endures objectification, nakedness, physical closeness, and unpleasant examinations.	He restricts interference from His parents. He receives help to avoid further embarrassment.
Theme 3	Being respectfully seen and cared for	He is met with interest and seen as a person. His difficulties are recognised and He feels that His worries are confirmed.	He is handled with care and attentiveness. His suffering is acknowledged.	He is comforted. The doctor’s explanation and plan are adapted to His needs, revealing that He is trusted and being taken seriously.
Theme 4	Navigating initiative and responsibility	He negotiates the right to set the agenda, define the problem, and be in charge of the discussion.	He is deprived of initiative and responsibility.	He negotiates His involvement in and responsibility for handling the problem and His future care.

#### Main theme: Struggling in no-man’s-land between childhood and adulthood

*Struggling in no-man’s land between childhood and adulthood* refers to the adolescent male’s attempts to gain the ability and sufficient support to handle the cognitive, verbal, and emotional challenges imposed by an encounter on unfamiliar terrain, while striving for responsibility and independence.

In the observed encounters, the adolescent male struggled to manage the encounter on His own, navigating between His will to take responsibility for His health and His need for support. He tried hard to make Himself understood and to understand what the GP was saying and what was happening. Due to His lack of experience of doctor’s appointments, He felt unsure about how to describe His symptoms, what to expect, and what was expected of Him. He endured feeling exposed, uneasy, and vulnerable in order to receive the help He required. He needed the doctor to provide clear explanations, comfort, and respect. He needed His parents’ protection when feeling vulnerable, as well as their help to understand, whilst this parental presence also entailed a risk of losing His right to define the problem and to take His part of the responsibility.

#### Theme 1: Being strained by incomprehensibility

*Being strained by incomprehensibility* means struggling to handle an important, difficult, and unfamiliar task without knowing how. Not knowing what is about to happen next, being expected to fulfil unknown expectations, and making sense of strange procedures or complicated explanations requires considerable effort, as does trying to find the words that will make the doctor understand.

Describing His problem required His full attention, concentration, and presence of mind. To help the doctor understand His problem, He had to provide all the necessary details as correctly and clearly as possible. Difficult questions had to be answered carefully. When at a loss, He would ask the doctor to clarify in order to clear up any potential misunderstandings.

Most of the time, the adolescent male prepared Himself by thinking through what to say or by reading up about His symptoms. At other times, His problems were self-explanatory, or He might choose to rely on His ability to spontaneously describe them or hope that a parent would speak on His behalf. There were even times when he was unaware of the reason for the visit.

Following the doctor’s sometimes rapid pace was challenging. Occasionally, the doctor would start talking before He had been properly seated, speak very quickly, or interrupt Him. At other times, the doctor facilitated His understanding by talking calmly, occasionally pausing to give Him time to think His answers through, and letting Him finish what He was saying. When the pace slowed down, He would talk more about His problem, either spontaneously or when encouraged by the doctor.

The adolescent male strived to fulfil the expectations of being a patient without knowing what this actually entailed. Sometimes He tried to convince the doctor of the severity of His problem by communicating with great intensity and vivid gestures; at other times, by appearing serious and composed. Inability to find a suitable answer to a question – or giving the wrong one – was a potential cause of shame. When the discussion turned to matters other than His problem, He could relax and perhaps even smile a bit.

Axel was obviously not at ease. He was fidgety, fiddling with His hands. He had to compose Himself before He started talking. He seemed prepared, but could not find the right words. He stopped, looking at His father for help. (…) The GP fired off a barrage of questions. Axel shifted in His chair, fidgeting with His glasses and with His hands. He laughed briefly and apologised for His behaviour.

During the examination, the adolescent male, not knowing what would happen next, dreaded embarrassment or pain. Often, understanding would relieve the tension. But even though the doctor typically explained what was happening throughout the examination, He might still be left confused when trying to interpret unfamiliar medical terms or comments such as ‘Looks good!’ in relation to His problem.

The GP rose abruptly from the chair. Nasir followed her with His gaze, watchful, attentive. The GP did not explain what she was about to do. Nasir waited quietly and cautiously.

In some cases, the GP left the room, only to return a moment later. During this hiatus, the adolescent male was partially and temporarily relieved from the strain of not understanding, as His more relaxed and spontaneous body language made evident.

In the explanation phase, understanding the doctor’s long, difficult, and sometimes muddled explanation required considerable effort. Complicated words were delivered to Him at a frenetic pace. Throughout this monologue, He was intent on assuring the doctor that He comprehended.

Perzy sat still, attentive. The GP explained. Perzy seemed keen to understand. The explanation was long and rather messy using complicated words, such as ‘otolaryngologist’. (…) Perzy frowned, leant forward, and concentrated hard.

Whenever He understood the doctor, He became less tense. He now appeared calm, relieved, and satisfied. He smiled, relaxed, and made more eye contact. The conversation slowed in pace. He was, however, exhausted, and as soon as He had understood (enough of) the doctor’s explanation and advice, He stopped listening, letting His parent take over the discussion or preparing to leave. However, if He had failed to make the doctor understand his problem, He would now have to be more explicit about His needs so as not to risk leaving without help. Even as the consultation came to a close, He might be *strained by incomprehensibility* in the sense of having to accept not being given an answer straightaway, having instead to endure uncertainty while waiting for further examinations, referrals, or spontaneous recovery.

#### Theme 2: Mitigating and enduring vulnerability

*Mitigating and enduring vulnerability* refers to the fact that disclosing thoughts, feelings and concerns to the doctor, as well as one’s body in a physical examination, may incur various degrees of embarrassment, defencelessness, and objectification. Limiting such distress is necessary to maintain one’s personal dignity.

As He described the problem, He struggled to maintain His dignity. Even when the doctor rushed Him, He took the time He needed to collect Himself before starting to talk. He answered clearly, in spite of sometimes having to censor His response to avoid revealing too much about Himself, His family, or His pets.

Carl was well prepared, knew what to say. He answered very clearly in a few words, as if watching His tongue. His arms were crossed, He looked tense (…) He rejected very firmly the suggestion that His cat could have been responsible for His symptoms.

Whenever these boundaries were transgressed, He would quickly adapt to the embarrassing situation, for instance by smoothing over whatever He had just said that prompted the doctor to pry too deep. In these situations, He might even appear at ease and untroubled. His parents could either exacerbate or alleviate His vulnerability. While being corrected by His parents was occasionally humiliating, they could also shield Him by shouldering the blame for failures or highlighting favourable aspects of Him.

*Mitigating and enduring vulnerability* raises questions about handling the pain, exposure, and disadvantage inherent in being the object of a physical examination. In the observed encounters, the doctor was in charge, whereas the adolescent male was typically quiet, restrained, and compliant. He endured the nakedness, physical closeness, and awkward, tickling, painful, or intrusive examinations that made Him uneasy. He expressed various degrees of attentiveness, watchfulness, or tension without necessarily being worried. When the doctor spoke to His parents while examining Him, He became more of an object than a person.

While examining Erik’s neck, the GP turned to Erik’s father, asking for his view of the problem.

The doctor generally recognised and limited the adolescent male’s vulnerability, for instance by letting Him keep as many of His clothes on as possible. Some parents also respected His privacy by stepping outside. Others protected Him by suggesting that the doctor omit painful examinations.

In the explanation phase, *Mitigating and enduring vulnerability* was largely about restricting parents who, in the adolescent male’s view, either interfered more than He preferred or embarrassed Him. Maintaining His dignity in these situations would be difficult without assistance. Some doctors helped avoid embarrassing situations by not presuming Him to have expensive equipment (e.g. a mobile phone for follow-up) or by asking for His permission before contacting His parents.

The GP told Ali that he would call Him in a few weeks and asked Him if He had a mobile phone of His own. The GP checked the number and asked permission to call Ali’s mother in case he would fail to reach Ali.

#### Theme 3: Being respectfully seen and cared for

*Being respectfully seen and cared for* is to be seen as a person and met with interest, attentiveness, and trust. This implies having one’s difficulties, needs, and feelings properly recognised and responded to through words and actions.

As He described His problem, the doctor showed genuine interest in His life. The doctor obviously tried to build rapport through kindness and an open, considerate, and friendly demeanour, but also appeared to see Him as a person. Sometimes they would laugh together. That He had some difficulties communicating was recognised without Him explicitly raising the issue. The doctor might, for example, stop talking about a subject that apparently embarrassed Him, or rephrase an explanation that He did not understand. He encountered interest, attentiveness and a will to help. The doctor took His problem seriously, asked follow-up questions, repeated, and clarified. His worries and suffering were verbally and emotionally confirmed, for instance by being assured that the appointment was necessary.

The GP leant forward, moved closer, slipped his glasses onto his forehead. He looked Ali in the eyes and spoke in a friendly tone.

During the examination, *being respectfully seen and cared for* is to be seen and treated considerately when vulnerable. Sometimes, He disclosed His anxiety; at other times, He was spared the trouble when the doctor, noticing His uneasiness, promised to be careful or apologised for the inconvenience. His suffering was recognised, such as when the doctor confirmed that His symptoms were reflected in the physical findings. Particularly meticulous examinations might appear ambiguous, either conveying a feeling of having been taken seriously or causing Him to worry about what was taking the doctor so long.

The GP confirmed for Majed that she saw that His skin was red and irritated. The GP listened intently to His heart and lungs. Did Majed feel cared for and taken seriously?

In the explanation phase, the doctor demonstrated trusting Him and taking Him seriously by basing their assessment on His symptoms and experience instead of questioning them. Many times, He was offered medication, further examinations, or follow-up. However, even though the doctor consistently spent a lot of time with Him, He would not always receive a clear answer.

His problems were acknowledged. The doctor comforted Him, eased His worries, and apologised when no quick fix was available. Difficulties in understanding the explanation were recognised and acted upon, such as when the doctor saw His confusion and explained once more. Sometimes, He was comforted and encouraged by His relatives as well.

The GP comforted Anton and told Him that he saw how ill Anton was, and that he (the GP) found it regrettable that he could not cure Anton. The GP asked how many pain killers He had taken.

#### Theme 4: Navigating initiative and responsibility

*Navigating initiative and responsibility* refers to the quest for balance between being the man who exerts His right to state the problem and takes responsibility for His health, the help-seeking patient with His intrinsic inferiority to the doctor, and the ill child who needs parental support.

In describing the problem, He was given the right to define it. The doctor listened attentively, gave Him time to explain what He meant, and checked to ensure that there were no misunderstandings. The doctor paid Him full attention, even if a parent or healthcare staff tried to interrupt. Sometimes He would take the initiative by interjecting while the doctor was speaking or by correcting the doctor’s misunderstandings. He might demonstrate responsibility for His own health by knowing His medical history, while His parents would remain quietly in the background until needed. When He did invite them into the discussion, e.g. to respond to a difficult question, they checked their views on the problem with Him before presenting them to the doctor.

Tomas knew what to say. He described His problems in a spontaneous manner. The story seemed not to have been prepared in advance. (…) The GP maintained a high pace and interrupted Tomas several times, but when she misunderstood Him, Tomas corrected her.

There were times when His parents took the initiative. Whenever this happened, He did not object. The parents might talk, uninvited, on His behalf or add supplementary details. Sometimes they interrupted or contradicted Him. Some stood close beside Him, supervising everything that happened; others suddenly stepped outside without asking His permission, for instance to answer a phone call. His parents could also take charge in more subtle ways, such as when their view of the problem was tightly intertwined with the adolescent males’ own description. Even absent parents could take the initiative by setting the agenda for the encounter when booking the appointment.

He allowed the doctor to take the lead in the encounter and steer the discussion without leaving Him much opportunity to describe what mattered to Him. In a subdued voice, He answered difficult questions elaborately, although some of them were of unclear relevance. He endured being interrupted by the doctor or having His mistakes corrected. He became more passive, accepting the doctor’s authority and His own powerlessness. If unaware of the reason for the encounter, He left it to the doctor to find out. The doctor could also demonstrate authority by firmly rejecting demands from parents or healthcare staff who tried to interrupt.

In the examination phase, the doctor had the initiative. He obeyed the doctor’s demands, even though the examination might be somewhat incomprehensible. However, He might temporarily take the initiative in order to, for instance, disclose that He was worried about painful procedures.

The GP examined Tomas. In a subdued voice Tomas told the GP that even the slightest touch caused Him pain.

In the explanation phase, the responsibility for His future care was negotiated. Crucially, the doctor might choose to explain things to Him or His parents. Although generally accepting the doctor’s responsibility for planning His care, being invited to discuss the plan would brighten Him up. Intent to take His part of the responsibility, He took care to demonstrate this by describing what steps He had taken towards self-care, presenting His plan for handling the problem Himself, or insisting on being the one to be called for follow-up. Sometimes, His parents would intervene to take back responsibility for the treatment or follow-up, at which point He would acquiesce, sometimes reluctantly, but at other times content with having received the answers that He needed.

The GP planned a follow-up by phone. Erik responded quickly and firmly that He wanted the GP to call Him, not His father. But Erik’s father intervened, suggesting that the GP call him instead, because Erik could not answer His phone while in school. Erik objected to this, but the GP accepted the father’s suggestion.

## Discussion

This is, as far as we know, the first video-observation study focusing on adolescent males’ encounters with GPs. Its most prominent finding was the complexity of these encounters. The adolescent male navigated between being an ill child in need of parental support and a man taking initiative and responsibility, while also fulfilling the unknown expectations of being a patient. Feeling exposed and vulnerable, He struggled to make Himself understood, and to understand what the GP said and what was happening. He needed to be seen with respect and to have His vulnerability recognised and carefully acted upon.

Exposing one’s vulnerability is an intrinsic aspect of being a patient [35]. In previous studies, adolescent males report difficulties in presenting themselves as vulnerable and help-seeking [3,36–38], fearing insufficient confidentiality [1,2,15,16] and being dismissed without help rather than being listened to [1,13,16]. What the present study adds is that adolescent males face several other difficulties, rendering them particularly vulnerable.

Using an approach novel to this context, the present study illuminates how adolescent males struggle to comprehend and express themselves, probably due to their unfamiliarity with encounters with GPs and their not yet fully developed neurocognitive abilities. It also elucidates how adolescent males strive to balance responsibility and support. They had to negotiate with the GP and their parents the right to define their problem and take responsibility for their own health, while also needing age-appropriate support in managing the encounter and their health. What is new is that this balancing act permeates the encounter. At one moment, support was needed; in the next, they strived for responsibility.

### Comprehensive understanding

Adolescent males’ encounters with GPs can be understood in relation to Danish philosopher K. E. Løgstrup’s thoughts on vulnerability in human encounters [39]. According to Løgstrup, it is inherent in human nature to approach others with trust. When a human being dares to come forward and be met, thereby trustingly exposing their vulnerability, an awareness of the ethical demand that is implied by receiving such trust arises. The demand is, in essence, to take care of what has been entrusted to one without using the power thus conveyed to intrude or encroach upon the other. In addition, the other’s individuality, will, and responsibility for their own life has to be respected. This is not the same as acting with superficial kindness, courtesy, or pandering to the wishes of the other; rather, it involves using one’s understanding of life to deem what best serves the other. When the ethical demand of receiving such trust is not responded to, but instead met with indifference or rejection, the individual’s embarrassment at having laid themselves bare for no purpose generates disappointment, a sense of being wronged, and distrust [39].

The medical problem that is put in their hands is often what makes GPs aware of the ethical demand [40]. In the present study, the GPs responded by addressing the problem in a serious, interested manner, being careful to understand what mattered to the adolescent males and how best to help. More importantly, the present study illustrates how a trusting adolescent male exposes His vulnerability, revealing His worries, His struggles to explain things well and to understand, as well as His inexperience and uncertainty as to what to expect or how to behave, and His shame when failing to fulfil expectations. The adolescent male’s vulnerability focuses the GP’s attention on the ethical demand, which entails much more than being kind. It is about taking care of His vulnerability in words and actions, such as having His confusion acknowledged, His worries comforted, and His embarrassment prevented or recognised. However, the GP can also fail to respond to the ethical demand, illustrated here as withholding emotional or compassionate reactions or abandoning the adolescent male to His lack of comprehension where He has to endure unintelligible questions delivered at a frenetic pace, dread unexplained examination procedures, or listen to opaquely worded explanations.

According to Løgstrup, to fully respond to the ethical demand, the GP should also serve the adolescent male while respecting His individuality, will, and responsibility for His own life. His individuality is respected by acknowledging Him as a person with a lifeworld of His own, but also – as the GP who draws upon their professionally-imbued understanding of life well knows – by taking His particular age-related needs into account [40]. Due to His young age, He may lack experience of healthcare and of His own bodily sensations, and may thus be unsure of what to expect and how to express His symptoms. Furthermore, since a person’s neurocognitive abilities are not considered to be fully mature until their mid-20s [41,42], He is still particularly vulnerable to stress, embarrassment, and other emotions, which can explain the great effort required to explain and to understand. Nevertheless, even when clearly demonstrating a will to take responsibility, His parents might intervene with a contrary view. The GP is thus presented with a dilemma where the parent’s behaviour can be interpreted either as intrusively depriving their son of His rightful responsibility for His health or as protecting Him from a responsibility that He wants, yet is unable to take. Overall, the present study illustrates the complexity of respecting adolescent males’ individuality through age-appropriate support while still respecting their will and responsibility in a more fundamental sense.

### Methodological considerations

In lifeworld research, there is never a single interpretation with an absolute claim to truth [22]. A valid interpretation fits both the whole and the details in the data, is consistent between the three interpretative arcs, and is more probable than other interpretations [23]. A valid interpretation also sheds new light upon the essential meaning of the studied phenomenon [22], in this case adolescent males’ lived experience of encounters with GPs. To achieve trustworthiness, the analysis moved back and forth between understanding and explanation in a process permeated by thoroughness, conscientiousness, dialogue, and critical questioning [20,22]. The authors strived for openness, curiosity, and sensitivity to the data to allow themselves to be surprised and have their preunderstandings challenged [23]. The interpretation was also checked for contradictions or omitted data [23]. The research process has been described in detail and excerpts are used to illustrate the structural analysis and ensure transparency.

#### Strengths and limitations

In the methodology of hermeneutic observations, a distinction is made between seeing and looking, where the former is more connected to capturing the deeper meaning of what is observed [20]. In the present study, the process of understanding was facilitated by the researchers being able to watch the video-recordings several times and discuss them together, each seeing the encounter through the lens of their own preunderstanding. The use of video-recorded observations also allowed analysis of facial expressions and body movements, which enabled us to elucidate the efforts made by the adolescent males in the encounters, the full extent of which would probably not have been revealed through interviews. The ongoing navigation between responsibility and support also became visible.

One possible concern about using video-recorded observations is that the procedure of recording a video might affect the participant’s behaviour. However, a structured literature review found no evidence in support of this hypothesis [43].

In three cases, the adolescent male remained seated in the visitor’s chair during the examination. Since all three were fully dressed during the examination, we deemed that the video-recordings could be used without compromising their integrity. In all other cases, analysis of the examination phase relied solely on audio, reducing its comprehensiveness somewhat.

A third limitation is that the understanding that one may gain from a hermeneutical observation study depends on the observers’ preunderstanding and their skill in capturing and expressing the meaning in the lived experience under study [20,32]. Three authors, JH, PK, and LJ, are GPs and thus well acquainted with the context. JH and PK are experienced in teaching medical students in consultation methods, and LJ is a bioethicist with experience of ethical observation studies. However, good knowledge of the field also entails a risk of being unable to see the phenomenon with fresh eyes [20]. To counter this limitation, LN and YT contributed with openness to the context. LN is a nurse, with long experience in qualitative research with previous experience of the phenomenological-hermeneutical method, phenomenological research, and hermeneutic observations. Finally, YT is a paediatrician with extensive experience in adolescent medicine, both in research and in the clinic.

Finally, the study only comprised nine consultations, which might be seen as a limitation. The nine encounters varied with regard to the adolescent males’ age, origin, reasons for visiting, and whether accompanied by a parent or not. As a result, the balance described between taking responsibility and the need for parental support is mostly based on the three consultations in which a parent was present, although absent parents sometimes also had an impact, for instance by setting the agenda. However, in phenomenological-hermeneutical studies, the number of observations needed depends on the study’s aim and the quality of the observations [20]. As the rather wide variation enhanced the richness of the data, the nine observations were deemed sufficient to reach information power [27]. The wide variation also enhances transferability to other contexts. Although transferability is for the reader to judge, we argue that the findings can deepen the understanding of adolescent males’ encounters with GPs in contexts similar to Swedish primary care, and that some parts may also be applicable in other types of healthcare encounters.

### Clinical implications

The unexpected complexity of a consultation with an adolescent male, even for relatively simple issues, imposes great demands on the GP who, in addition to managing the medical problem, has to recognise and adapt to the patient’s individual needs. The GP may fail to respond to these challenges due to time constraints, lack of knowledge, or by being overwhelmed by the complexity of the situation [44]. In such cases, the adolescent male may feel embarrassed at having disclosed things about Himself and His concerns in vain and possibly come to distrust the GP. We suggest that this is one rather plausible explanation for adolescent males’ reports of negative experiences, in spite of GPs’ patient-centred intentions. To properly respond to the ethical demand, the GP should endeavour to be as attentive and adaptive as possible, strive to note and respond to any signs of pain or embarrassment, and provide ample emotional validation, preferably verbally through phrases such as ‘That must be hard for you’ [45].

To bridge the gap between inexperience and immaturity, GPs may want to adopt the suggestions that adolescent males themselves provided in a previous study: keep the pace unhurried, clearly convey what is going to happen, and phrase the medical assessments in plain language [46]. Notably, we observed that when the pace slowed down, the adolescent males were better able to elaborate on their issues, and when they appeared to understand the GP’s explanations, their tension eased. We recommend that GPs pay close attention to signs indicating that the adolescent male may not fully understand, and that the GPs verify that they have understood by inviting the adolescent male to correct any misunderstandings by the GP.

To help the adolescent male balance between independence and support, the GP may provide time alone in private, i.e. without accompanying parents. However, as the needs of an adolescent male may vary from one minute to the next, providing private time might not be sufficient. We therefore suggest that the GP pay attention to this duality in order to support the adolescent males’ growing autonomy, as well as promoting wise health decisions. This can be done by emphasising His right to take initiative, for instance by encouraging Him to define the problem and participate in decisions regarding His health, and by helping Him regain this right when His parents (or the GP themselves) are too eager to take charge.

## Conclusion

Adolescent males face several complex difficulties in encounters with GPs. They struggle to navigate the challenges of unfamiliarity, cognitive demands, vulnerability, and balancing responsibility and support. When an adolescent male reveals things about Himself and His concerns, the GP faces an ethical demand to take care of what is put in their hands. To respond to that demand, the GP should use their understanding to support the adolescent male in navigating the encounter by respecting Him as a person and meeting His age-dependent needs. There can be no hard and fast rules here; instead each GP must in every case judge how to serve Him best. These complex encounters thus impose great demands on the GP, which may explain why, despite their good intentions, they may fail to fully take care of all that is laid in their hands. Future studies can preferably explore how GPs experience these kinds of encounters and the demands placed upon them, as well as what they actually do when successfully serving adolescent males’ needs.

## Data Availability

The authors would like to thank all participants and the staff at the two participating healthcare centres. To protect the participants’ privacy, the video recordings are not publicly available, but are available from the corresponding author upon reasonable request.
